# To explore the reasonable selection of clavicular hook plate to reduce the occurrence of subacromial impingement syndrome after operation

**DOI:** 10.1186/s13018-021-02325-5

**Published:** 2021-03-09

**Authors:** Rui Qiao, Jiarui Yang, Kun Zhang, Zhe Song

**Affiliations:** 1 Department of Orthopaedic Trauma, Hong Hui Hospital, Xi’an Jiaotong University School of Medicine, Xi’an, 710054 Shaanxi China; 2 Xi’an Medical University, Xi’an, 710054 Shaanxi China

**Keywords:** Clavicular hook plate, Acromion impact sign, Dislocation, Acromioclavicular joint

## Abstract

**Background:**

Acromioclavicular joint dislocation is a shoulder joint injury common in the clinical setting and is generally surgically treated with clavicular hook plate technique with confirmed curative effect. However, symptoms such as shoulder abduction limitation, shoulder discomfort and joint pain postoperatively may occur in some patients. Therefore, this study aimed to explore whether the existing clavicular hook plate can be reasonably selected to reduce the incidence of subacromial impingement syndrome (SIS) and provide a reference for clinical diagnosis and treatment.

**Materials and methods:**

Patients with SIS admitted from March 2018 to June 2020 were selected as the experimental group and asymptomatic patients postoperatively, as the control group. The hook end depth and acromial height of the hook plate used in patients were recorded, and the difference between them was calculated.

**Results:**

The difference between the hook plate depth and acromial height was 7.500±1.912 mm and 6.563±1.537 mm in the experimental and control groups, respectively, with statistically significant difference (*t*=3.021, *P*=0.006). A difference of >0.6 mm as a grouping index is required to perform a single factor analysis, with statistically significant difference (*t*=3.908, *P*=0.048).

**Conclusions:**

The occurrence of SIS after placing the clavicular hook plate may be related to the difference between its depth and the acromial height. A difference of >6 mm may be a factor affecting the occurrence of SIS. Pre-imaging measurement of the acromial height can provide suggestions for selecting the type of hook plate intraoperatively.

## Background

Acromioclavicular joint dislocation is a common shoulder joint injury in the clinical setting and is generally surgically treated with clavicular hook plate technique with confirmed curative effect. However, symptoms such as shoulder abduction limitation, shoulder discomfort and joint pain postoperatively may occur in some patients [1, 2]. After the second surgery to remove the hook plate, the shoulder discomfort is thought to be relieved or even disappear [3]. However, their quality of life was severely diminished before the hook plate removal. Most scholars believe that the shape of the acromion is poorly matched with the currently used steel plate, causing an acromial impact sign, i.e. subacromial impingement syndrome (SIS) after the hook plate surgery [4, 5]; however, designing a fully fitted hook plate is difficult due to individual differences. Therefore, this study retrospectively analysed patients with SIS from March 2018 to June 2020 to explore whether the existing clavicular hook plate type can be reasonably selected and used in order to reduce the occurrence of SIS and provide a reference for clinical diagnosis and treatment.

## Methods

Inclusion criteria are as follows: patients (1) with acromioclavicular joint dislocation and surgical treatment with clavicular hook plate, (2) aged >18 and <60 years, (3) with fresh closed acromioclavicular joint dislocation within 2 weeks, (4) who were followed up for >8 months and (5) with good compliance. Exclusion criteria are as follows: patients with (1) fracture in other body parts; (2) osteoporosis; (3) pathological fracture; (4) serious internal diseases, tumour or mental illness without functional exercise; (5) poor ligament healing found on MRI 8 months postoperatively and (6) history of shoulder disease. From March 2018 to June 2020, a total of 170 patients met the inclusion criteria: 34 with SIS were considered the experimental group and 136 without SIS, the control group. The experimental group comprised 23 men and 11 women, with an average age of 21.647±6.440 (range, 19–48) years: 10 on the left and 24 on the right. The control group consisted of 85 men and 51 women, with an average age of 34.140±9.139 (range, 21–54) years: 55 on the left and 81 on the right. No significant difference in sex, age or position was observed between the two groups (*P* >0.05) (Table 1).

**Table 1:** Patient data

Variable	Experimental group (*n* = 34)	Control group (*n* = 136)	Test statistics	*P* value
Age (year)	31.647±6.440	34.140±9.139	1.499	0.136
Gender (male/female)	23/11	85/51	0.311	0.577
Position (left/right)	10/24	55/81	1.401	0.237

### Follow-up

**Fig. 1:**
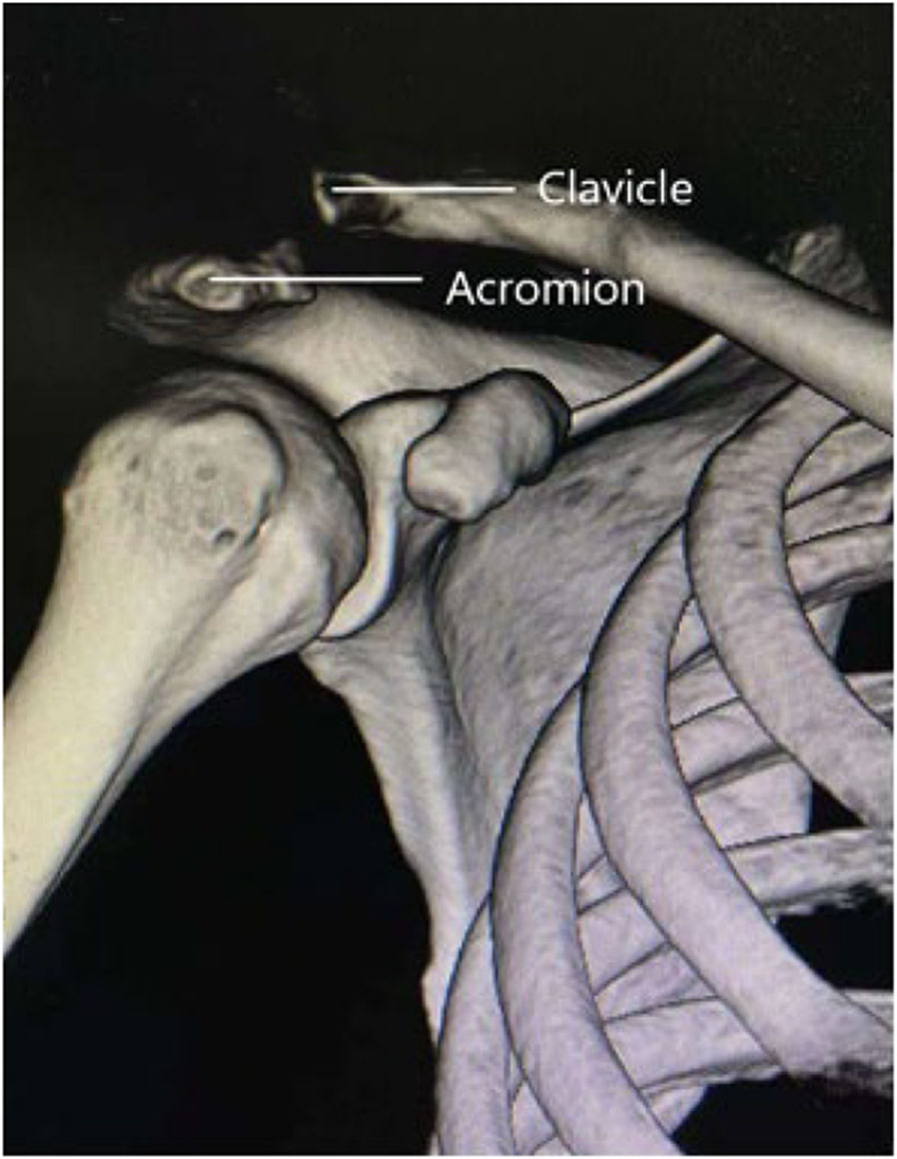
Preoperative CT image of a 32-year-old man who fell and sustained a right acromioclavicular joint dislocation

**Fig. 2:**
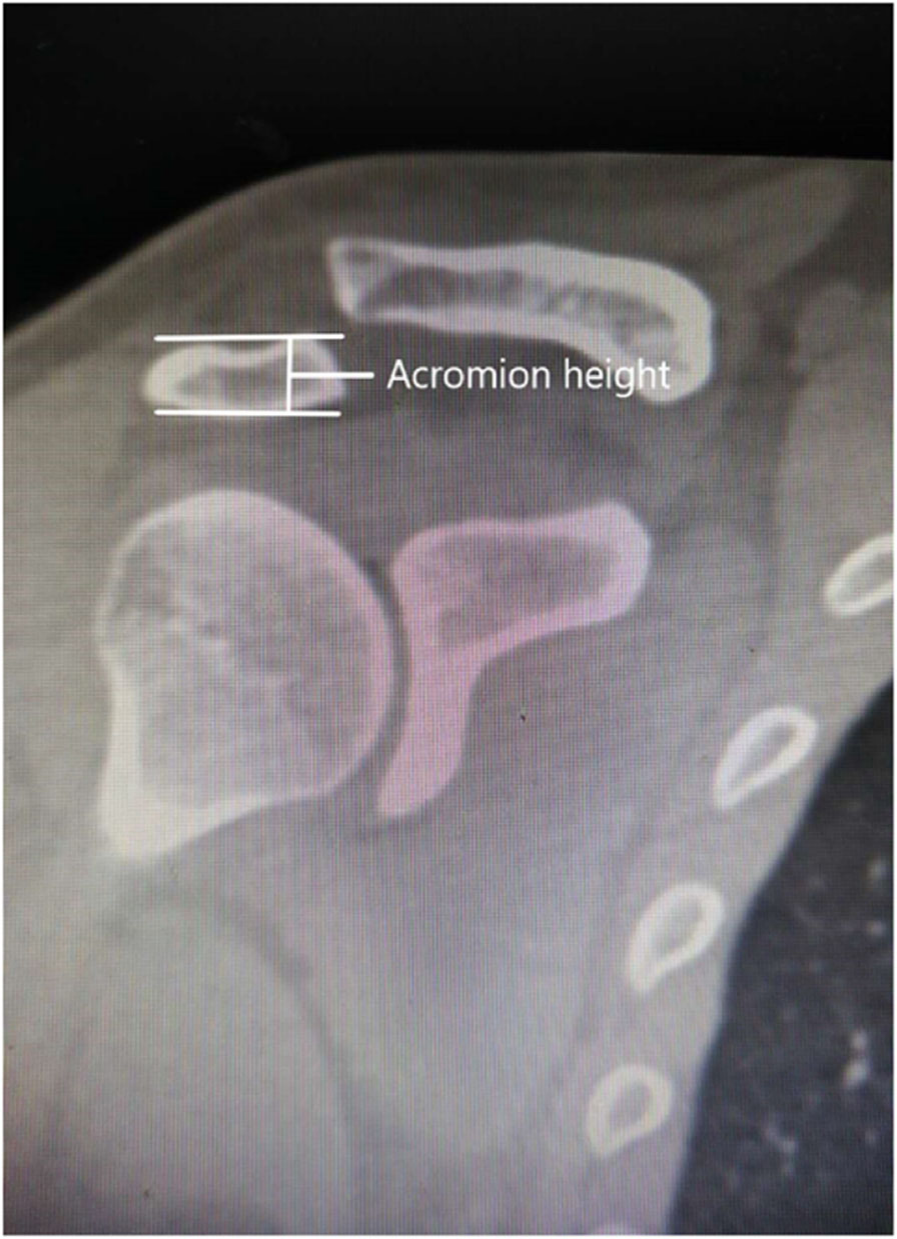
Measurement of the acromial height on coronal plane CT

**Fig. 3:**
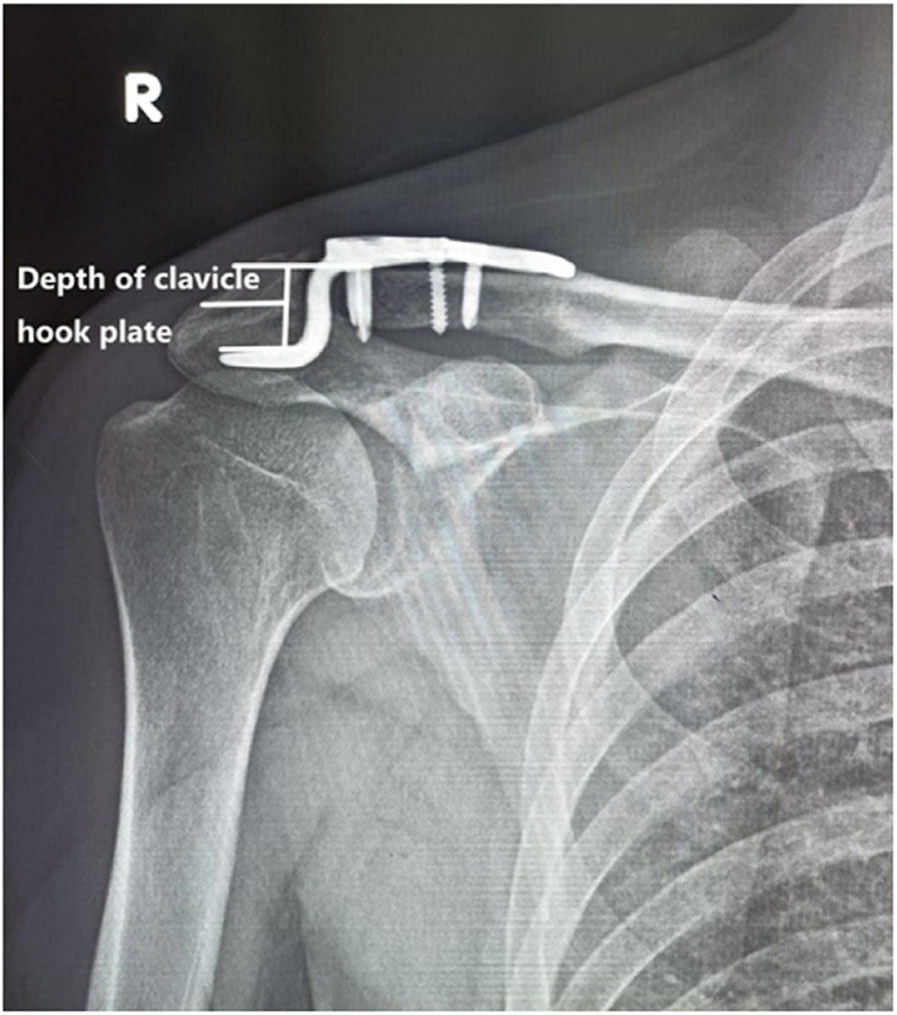
The depth of the hook plate used in the patient

Preoperatively, spiral CT was used to examine and diagnose patients (Fig. 1). The acromial height was measured on the coronal plane, that is, the thickest acromion on the coronal plane (Fig. 2). Then, the hook end depth (Fig. 3) and the acromial height were recorded, their differences were calculated. The clavicular hook plate was selected from Tianjin Zhengtian Company, with length of 20 mm, width of 6 mm and thickness of 3 mm. The clavicular hook plate is classified into three depths: 12 mm, 15 mm and 18 mm.

Postoperatively, the upper arm was suspended and fixed, and the shoulder pendulum training was started immediately after the anaesthesia dissipated. After 2 weeks, the sling was removed, and the patient was instructed to start unrestricted joint motion training. Patients were followed up for 2 weeks, 1, 3, 6 and 8 months postoperatively. The rehabilitation plan was established according to the patient’s recovery status. After 8 months, if symptoms, such as limited shoulder joint abduction, shoulder discomfort and joint pain, and positive Neer impact and Hawkins impact signs were observed, the patient was diagnosed with SIS after excluding hook plate dislocation, stress fracture and other diseases.

### Statistical analysis

The Statistical Package for Social Sciences software version 19 (IBM, Chicago, IL, USA) was used. Data are presented as means and standard deviations, except when noted. *c*^2^ and Fisher’s exact tests were performed for categorical variables as appropriate. Comparisons between intergroups were performed using Student’s *t* test for continuous variables.

## Results

The acromial height was measured using the CT coronal plane: 8.294±0.802 mm and 8.614±1.092 mm in the experimental and control groups, respectively, without statistically significant differences (*t*=1.598, *P*>0.05). In the experimental group, 14 (41.18%), 15 (44.12%) and 5 (14.71%) patients were prescribed a depth of 18 mm, 15 mm and 12 mm, respectively. In the control group, 39 (28.68%), 66 (48.53%) and 31 (22.80%) patients were prescribed a depth of 18 mm, 15 mm and 12 mm, respectively. No significant difference in the type of steel plate used was observed between the two groups (*t* = 1.499, *P* > 0.05); however, the proportion of steel plates with 18 mm specification was found to be lower in the control group than that in the experimental group. The difference between the hook plate depth and acromial height was 7.500 ±1.912 mm and 6.563 ±1.537 mm in the experimental and control groups, respectively, with statistically significant difference (*t* = 3.021, *P*=0.006). Therefore, the difference between the hook depth and acromial height is extremely large, and SIS may be more likely to occur. Finally, univariate analysis was carried out with different values of >5.5 mm, >6.0 mm, >6.5 mm, >7 mm, >8 mm, >9 mm and >10 mm as grouping indices (Table 2). No significant difference was observed in values of >5.5 mm (*t* = 7.446, *P* = 0.060). Other groups showed statistically significant differences; therefore, when the difference between the hook plate depth and acromial height is reported to be >6 mm, patients may be more likely to develop SIS postoperatively.

**Table 2:** Univariate analysis of factors influencing acromion impingement syndrome

Variable	Experimental group (*n* = 34)	Control group (*n* = 136)	Test statistics	*P* value
*D* value				
>5.5 mm	30	99	3.544	0.060
≤5.5 mm	4	37		
>6 mm	28	88	3.908	0.048
≤6 mm	6	48		
>6.5 mm	26	75	5.129	0.024
≤6.5 mm	8	61		
>7 mm	22	55	6.463	0.011
≤7 mm	12	81		
>8 mm	13	18	11.402	0.001
≤8 mm	21	118		
>9 mm	9	9	11.324	0.001
≤9 mm	25	127		
>10 mm	4	1	11.591	0.001
≤10 mm	30	135		

## Discussion

A clavicular hook plate is designed according to the shape of the human acromioclavicular joint, and the reduction of the acromioclavicular joint is determined using the lever principle [6]. The risk of postoperative plate fracture is low. Due to its simple procedure and good curative effect, the clavicular hook plate is widely used in the clinical setting. However, a series of complications can occur after the clavicular hook plate surgery [7]: (1) failure of internal fixation and poor ligament repair; (2) occurrence of postoperative SIS symptoms, such as periacromial pain, upper limb weakness and limited shoulder joint abduction; (3) subacromial osteolysis and (4) clavicular stress fracture and acromial stress fracture.

The incidence of SIS after the clavicular hook plate surgery is 19–25% [8, 9]. Therefore, researchers have continued to explore the causes of SIS after clavicular hook plate surgery. Macdonald et al. [10] thought that the occurrence of SIS after the clavicular hook plate surgery was related to the acromion shape and curved and hooked acromion were more likely to cause SIS than straight acromion. Several studies [2, 9, 11] have shown that older patients who underwent hook plate surgery have increased risk of SIS, which may be related to shoulder tissue degeneration. Macdonald et al. [12] found that the placement of the clavicular hook plate would inevitably narrow the subacromial clearance, which may be related to the occurrence of SIS. Elmaraghy et al. [13] simulated clavicular hook plate implantation on cadavers and found that the occurrence of SIS was related to reduced subacromial space, but not to the angle of hook plate implantation. However, multiple reasons may cause the occurrence of SIS postoperatively. We hope that the appropriate hook plate can be selected to reduce the possibility of SIS. When analysing the reasons, the difference between the hook end depth and acromial height will be too large when the type of hook plate used by the patient does not match with that of the patient itself, resulting in reduced subacromial space. When the hook end occupies too much space, the patient’s shoulder abduction will cause pain because the hook plate squeezes the subacromial tissue such as the rotator cuff and subacromial subdeltoid bursa [14]. Therefore, when selecting hook plates intraoperatively, we should avoid choosing models with a large acromial height difference with that of the patient to prevent hook plates from occupying too much subacromial space and reduce the occurrence of postoperative SIS.

Many scholars have provided suggestions on how to prevent the occurrence of SIS after the hook plate surgery. Li Kui et al. [15] suggested using shoulder arthroscopy to examine whether the hook plate occupies the acromion space intraoperatively and then adjust the hook plate type. Li-Kun Hung et al. [16] simulated the implantation process of clavicular hook plates at different hook angles (90°, 95°, 100°, 105° and 110°) for the treatment of acromioclavicular joint dislocation and found clavicular hook plates with different hook angles, causing different biomechanical behaviours of the clavicle and acromion. Using implant clavicle hook plates with different hook angles according to different shoulder joint shapes is recommended. Yin JP et al. [4] conducted morphological studies on the clavicular hook plate and acromioclavicular joint and found that the occurrence of SIS was related to the mismatch between the clavicular hook plate and acromioclavicular joint, suggesting a personalised use of clavicular hook plate. However, due to the influence of intraoperative patient position, X-ray acromial imaging remains inaccurate [17]; however, promoting the use of shoulder arthroscopy during the intraoperative assessment of the subacromial space is difficult. Therefore, the space occupied by the hook plate in the subacromial space cannot be measured intraoperatively, thus, not providing adequate information for surgeons to select the hook plate type. Due to individual differences of acromion shape, a personalised clavicular hook plate cannot be created. We believe that the acromial height can be measured by CT preoperatively, and the difference between the end depth of the hook plate and the acromial height should be controlled within 6 mm to reduce the occurrence of postoperative SIS.

**Fig. 4:**
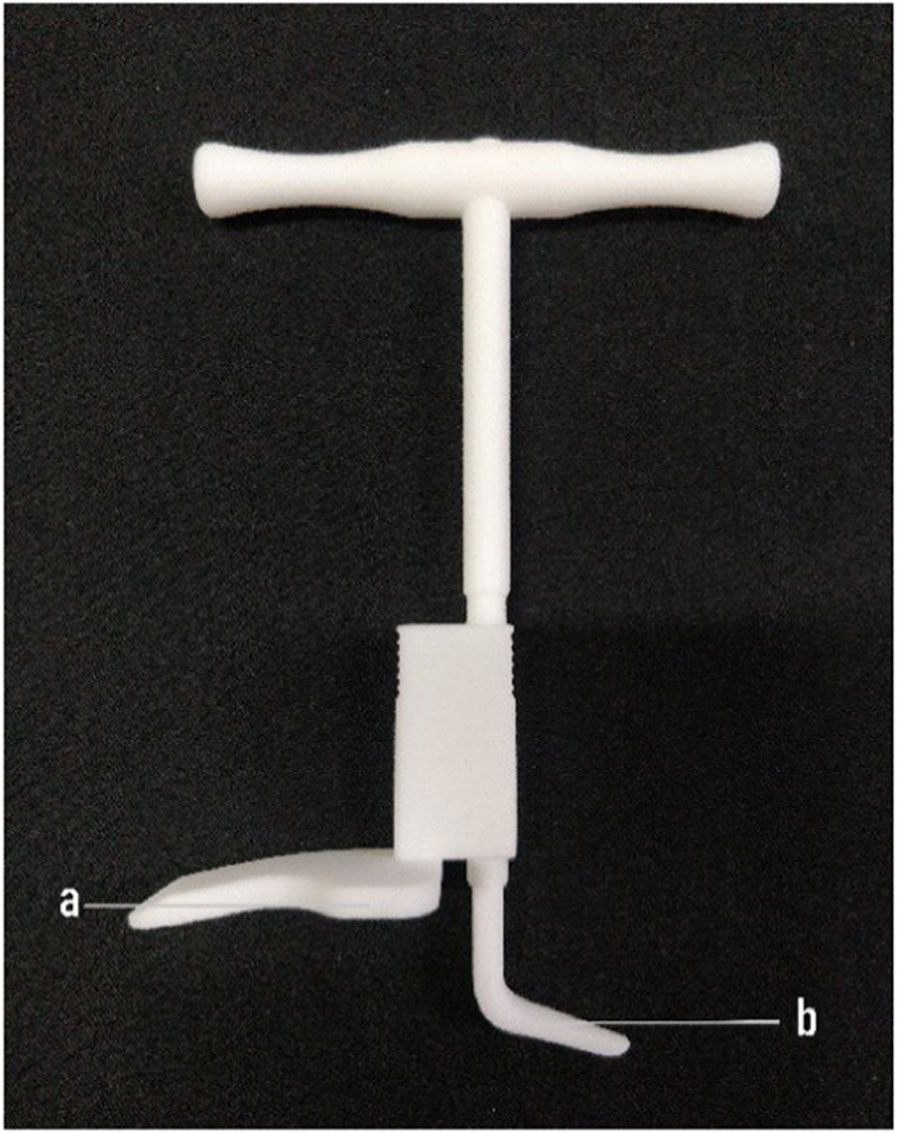
The acromial height measuring device

Preoperative imaging measurement of the acromial height helps select the type of hook plate used intraoperatively; however, data on the acromion may be inaccurate due to the presence of CT bone artefacts and cartilage. Therefore, the surgeon should measure the acromial height intraoperatively and select the appropriate clavicular hook plate. Our team developed a device to measure the acromial height intraoperatively (Fig. 4).

**Fig. 5:**
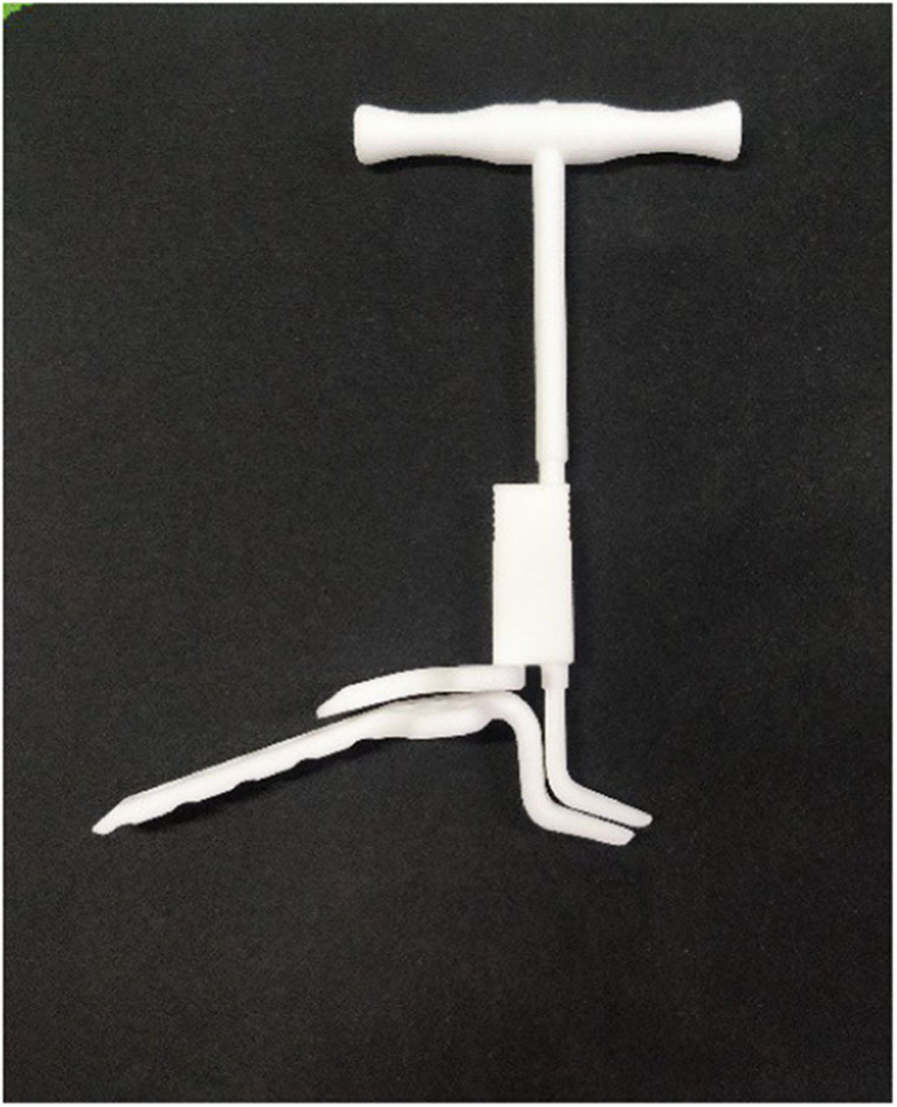
Acromial height measuring the instrument and clavicular hook plate

The shape of the measuring instrument matches that of the currently used hook plate (Fig. 5). The acromioclavicular joint is exposed and reduced intraoperatively; the side is placed at the distal end of the clavicle and the b side is placed under the acromion. The acromial height was measured by rotating the handle. Then, the appropriate steel plate is selected to reduce the occurrence of postoperative SIS. At present, the measuring instrument is already in production, and its effect should be further studied after being used in the clinical setting.

## Conclusions

In summary, the occurrence of SIS after the clavicular hook plate surgery may be related to the difference between the hook plate depth and acromial peak height. A difference of >6 mm may be an influential factor for the occurrence of SIS. The measurement of acromial height by imaging preoperatively can provide information for the selection of the hook plate model intraoperatively; however, defects may exist. Therefore, the use of an acromial height metre is expected in clinical practice.

## Data Availability

All data generated or analysed in this study are included in this published article.

## Abbreviations

SIS: Subacromial impingement syndrome
